# Tape suture for stabilization of incomplete posterior pelvic ring fractures—biomechanical analysis of a new minimally invasive treatment for incomplete lateral compression pelvic ring fractures

**DOI:** 10.1186/s13018-019-1509-y

**Published:** 2019-12-27

**Authors:** Christopher Alexander Becker, Adrian Cavalcanti Kussmaul, Eduardo Manuel Suero, Markus Regauer, Matthias Woiczinski, Christian Braun, Wilhelm Flatz, Oliver Pieske, Christian Kammerlander, Wolfgang Boecker, Axel Greiner

**Affiliations:** 1 Department of General, Trauma and Reconstructive Surgery, University Hospital, LMU Munich, Munich, Germany; 2 Department of Orthopedic Surgery, Physical Medicine and Rehabilitation, University Hospital, LMU Munich, Munich, Germany; 3 Department of Legal and Forensic Medicine, University Hospital, LMU Munich, Munich, Germany; 4 Department of Radiology, University Hospital, LMU Munich, Munich, Germany; 5 Department of Trauma Surgery, Evangelic Hospital Oldenburg, Oldenburg, Germany

## Abstract

**Background:**

Incomplete lateral compression fractures (including AO Type B2.1) are among the most common pelvic ring injuries. Although the treatment of choice remains controversial, sacroiliac (SI) screws are commonly used for the operative treatment of incomplete lateral compression fractures of the pelvic ring. However, the disadvantages of SI screws include the risk of nerve root or blood vessel injury. Recently, tape sutures have been found useful as stabilizing material for the treatment of injuries of the syndesmosis, the rotator cuff and knee ligaments. In this current study, we aimed to test the biomechanical feasibility of tape sutures to stabilize the pelvis in the setting of AO Type B2.1 injury.

**Methods:**

Six human cadaveric pelvises underwent cyclic loading to compare the biomechanical stability of different osteosynthesis methods in a B2.1 fracture model. The methods tested in this experiment were a FiberTape® suture and the currently established SI screw. A 3D ultrasound tracking system was used to measure fracture fragment motion. Linear regression was used to model displacement and stiffness at the posterior and anterior pelvic ring.

**Results:**

At the posterior fracture site, the FiberTape® demonstrated similar displacement (2.2 ± 0.8 mm) and stiffness (52.2 ± 18.0 N/mm) compared to the sacroiliac screw (displacement 2.1 ± 0.6 mm, *P* >  0.999; stiffness 50.8 ± 13.0 N/mm, *P* > 0.999).

Considering the anterior fracture site, the FiberTape® again demonstrated similar displacement (3.8 ± 1.3 mm) and stiffness (29.5 ± 9.0 N/mm) compared to the sacroiliac screw (displacement 2.9 ± 0.8 mm, *P* = 0.2196; stiffness 37.5 ± 11.5 N/mm, *P* = 0.0711).

**Conclusion:**

The newly presented osteosynthesis, the FiberTape®, shows promising results for the stabilization of the posterior pelvic ring in AO Type B2.1 lateral compression fractures compared to a sacroiliac screw osteosynthesis based on its minimal-invasiveness and the statistically similar biomechanical properties.

## Introduction

Fractures of the pelvic ring are frequently seen, especially in polytrauma patients or geriatric patients with poor bone quality [1–3]. Incomplete lateral compression fractures (including AO Type B2.1) are among the most common pelvic ring injuries [4, 5]. The standard treatment of the incomplete lateral compression pelvic ring fracture is controversially discussed, ranging from conservative treatment to surgical care [5, 6]. As a standard concept, the sacroiliac screw (SI screw) fixation is part of the operative care and has shown sufficient biomechanical stability [7–9]. But the disadvantages of this method include nerve root and/or blood vessel damage in case of misalignment of the screw [9, 10]. If two sacroiliac screws are used (S1 and S2), there is an even higher risk of damaging the nerve roots S1/S2 with the second S2-screw [11]. Furthermore, the necessity to compromise the intact sacroiliacal joint in the process of fixing the lateral sacral fracture is questionable. Regarding this problem, a variety of operating methods have been recently developed to stabilize sacral fractures [2, 9, 12, 13].

Recently, tape sutures have proven to be a stabilizing method for syndesmotic injuries in the ankle joint, for injuries to the rotator cuff of the shoulder or for ligamental knee injuries [14–16]. Similar to the recently described transiliac internal fixator (TIFI), which is installed to the posterior superior iliac spine, one could insert a tape suture through this anatomical structure to minimal-invasively stabilize the posterior pelvic ring [12, 13, 17].

The disadvantage of the transiliac internal fixator lays in the possible disturbance of the soft tissue structures dorsal of the posterior superior iliac spine [17]. By using a tape suture one could possibly avoid this issue. Another advantage of a tape suture represents the higher flexibility of the tape compared to the rigidity of a screw. In ankle surgery, a tape suture performs a semi-rigid transfixation of the tibiofibular joint allowing micromotions in movement [18].

In this study, we assume that a tape suture will perform the semi-rigid transfixation of both sacroiliac joints. In addition, when inserting a tape suture in the posterior superior iliac spines, no relevant nerve structure is endangered.

For this reason, we performed a biomechanical analysis on 6 fresh frozen cadaver pelvises to analyze the stability of the tape suture applied to the posterior pelvic ring compared to a sacroiliac screw.

## Material and methods

### Material

A total of 6 human cadaver pelvises collected between January 2016 and April 2017 were used in this study with the approval of our institution’s ethics committee and the approval of the donors’ relatives given prior to the experiments. Only intact pelvises without any preexisting damage to the musculoskeletal structure, a tumor, or tuberculosis disease was included in this study. The characteristics of the pelvises are displayed in Table 1.

**Table 1:** Characteristics of the specimens

Pelvis	Age (years)	Sex	BMD (mg Ca-Ha/ml)
1	74	Male	104.6
2	72	Male	113.7
3	25	Male	151.7
4	67	Male	63.2
5	60	Male	121.6
6	65	Male	133.7
Mean	60.5 ± 18.1		114.8 ± 30.1
Median	66		117.7

### Methods

Prior to any processing, the bone density of all pelvises was measured with a qCT scan using the fourth and fifth lumbar vertebrae (Table 1).

The pelvises were unfrozen 1 day prior to their experiment. On the day of the experiment, each pelvis was heated in a water bath at roughly 35 °C for 30 min in order to approximate body temperature.

**Fig. 1:**
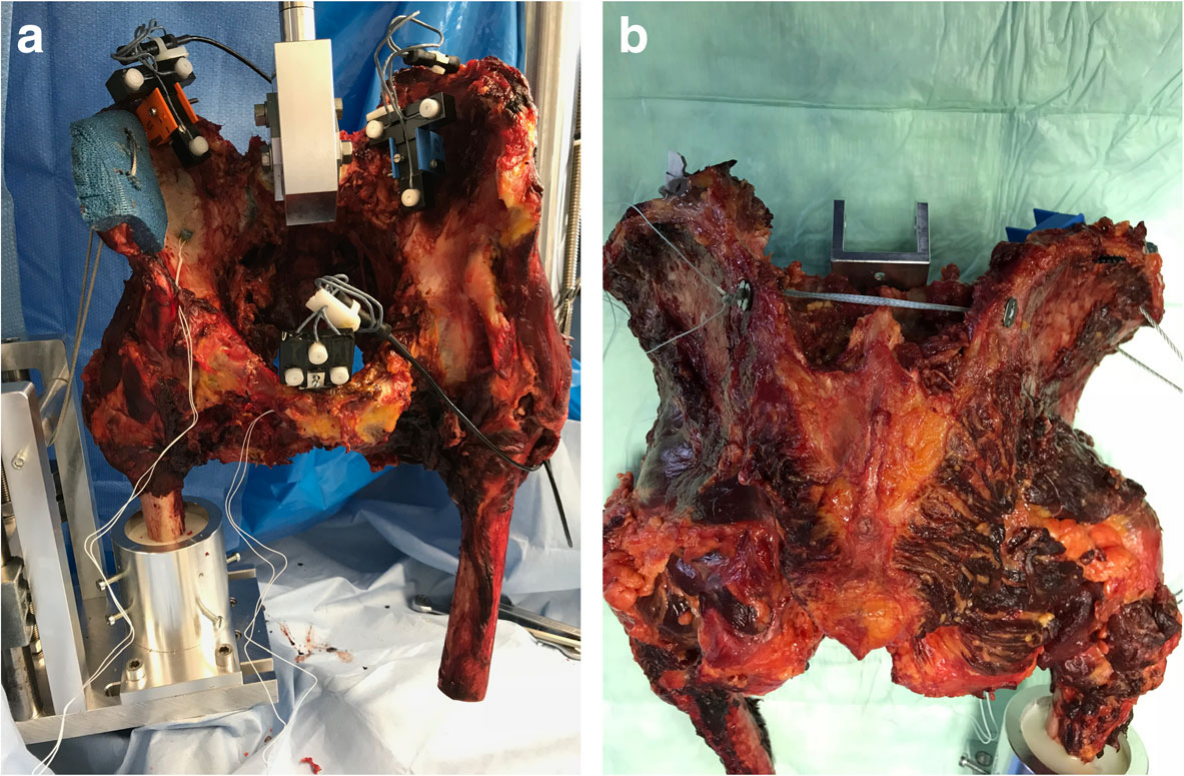
**a** Positioning of the pelvis onto the testing rig. **b** FiberTape® spanning the posterior superior iliac spines

Once heated, the tissue covering the spots needed to install the experiments equipment (Fig. 1a) was dissected; however, the dissection was kept as little as possible to preserve ligamental structures.

After the removal of the tissue, each pelvis was mounted on the testing machine and the 5-step protocol (Table 2) was applied. The first test served as a reference measurement of the intact pelvis (Trial 1: ‘reference’). Secondly, an AO type B2.1 fracture was created in accordance with the AO classification, consisting of a partial sacral fracture reaching from the superior margin of the sacrum until the height of the second sacral neuroforamen and of the anterior fracture consisting of an ipsilateral anterior pelvic ring fracture.

**Table 2:** Test protocol

Step 1	Loading up to 150 N
Step 2	Holding at 150 N for 30 s
Step 3	Periodic loading: 20 cycles with a frequency of 0.25 Hz between 150 N and 250 N
Step 4	Holding at 250 N for 135 s
Step 5	System back to its original position of + 28 mm

Next, the fixation methods were applied with the either tape suture (FiberTape 2 mm, Arthrex, Naples, FL, USA) spanning between both posterior superior iliac spines (Fig. 1b) or the 6.5 mm × 70 mm cannulated, partially threaded sacroiliac screw (DePuy Synthes, Umkirch, Germany) being inserted into the first sacral vertebrae (S1).

For the insertion of the tape suture (FiberTape®), two osseous channels were drilled through both posterior superior iliac spines, beginning from the fracture site using a high speed drill. The drill went through the contralateral posterior superior iliac spine from medial to lateral to achieve an osseous channel in the same direction as on the ipsilateral site. The FiberTape® was then threaded through the bony holes and a metal washer (DogBone®-Button, Arthrex, Naples, FL, USA) first on the contralateral site. Both ends were pulled back through the osseous channel with the tape consequently spanning between both iliac spines. The ipsilateral ends were then threaded through a second DogBone®-Button and manually surgically tied. Consequently, both metal washers were lying on the lateral sides of the osseous channels on each iliac spine (Fig. 1b).

For the S1 screw, the following technique was used. The S1 partially threaded screw was inserted under fluoroscopic control on the fractured site using the S1-corridor above the first neuroforamen, with at first drilling of a Kirschner-wire and secondly inserting the screw using the previously drilled K-wire.

Finally, the pelvises were set up in the testing machine.

### Methods of assessment

In this study, we used an all-electric testing machine (Instron ElectroPulsTM E10000 Linear-Torsion, Norwood, MA 02062-2643, USA) and a 3D-ultrasound measuring system (Zebris CMS20, Gilching, Germany) for the recording of all data.

The ultrasound system consisted of 3 sensors that were placed onto the pelvis as seen in Fig. 1 and a transducer that was positioned 50 cm anteriorly to the pelvis. The unilateral embedding of the femur into a metal cylinder containing epoxide resin allowed the simulation of a single leg stance while the superior clamp dissembled a spherical joint.

For the conduction of the experiment, a 5-step protocol was applied according to McDonald et al. [19] (Table 2).

Every 30 ms, the position of all sensors was recorded, allowing the calculation of the relative distances between the sensors at any time. These distances were then used for the statistical analysis.

### Statistical analysis

We used linear regression to model the magnitude of displacement between the fracture fragments representing the functionality of the stabilizing techniques used: native pelvis (reference), SI screw, and FiberTape®. Clustered standard errors were calculated using the Huber-White method. Pairwise comparisons were carried out using *t* tests. The Bonferroni method was used to adjust the *P* values for multiple comparisons. For all tests, *α* was set to 0.05. Descriptive statistics are presented as mean ± standard deviation (SD) wherever appropriate.

## Results

**Fig. 2:**
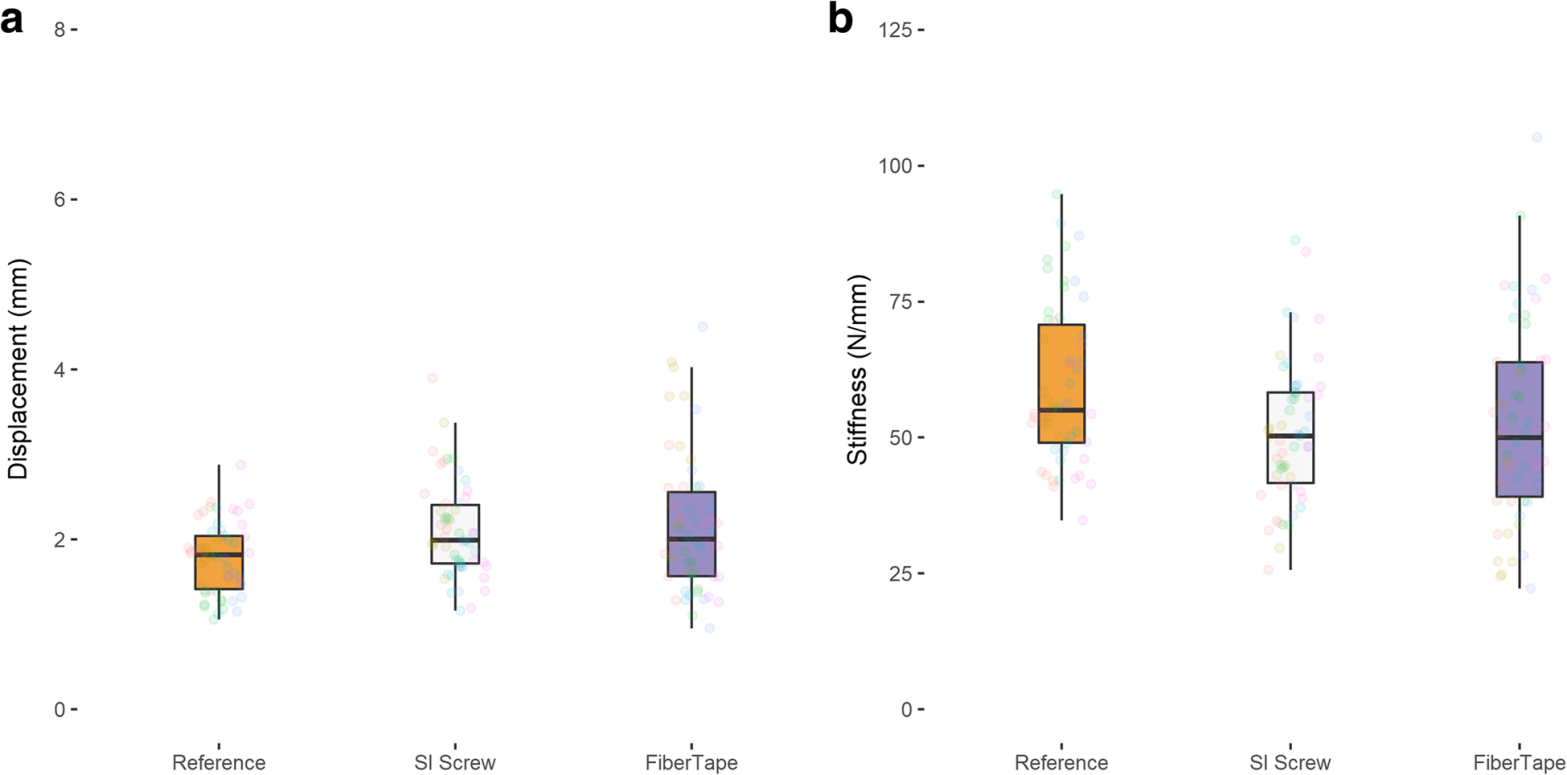
**a**, **b** Average displacement and stiffness measurements at the posterior fracture site

At the posterior fracture site, the FiberTape® demonstrated similar displacement (2.2 ± 0.8 mm) and stiffness (52.2 ± 18.0 N/mm) compared to the sacroiliac screw (displacement 2.1 ± 0.6 mm, *P* >  0.999; stiffness 50.8 ± 13.0 N/mm, *P* >  0.999) (Table 3) (Fig. 2).

**Table 3:** Mean displacement and stiffness measurements for pelvic fractures fixed with either an SI screw or with FiberTape. The *P* value shown is for the difference between both groups

		Reference	FiberTape®	Sacroiliac screw	*P* value
Mean displacement (mm)	Posterior	2.3 ± 0.5	3.2 ± 0.9	3.0 ± 0.5	> 0.999
	Anterior	2.2 ± 0.4	4.5 ± 1.5	3.6 ± 0.6	0.2196
Mean stiffness (N/mm)	Posterior	44.2	31.7	32.8	> 0.999
	Anterior	46.5	24.1	28.9	0.0711

**Fig. 3:**
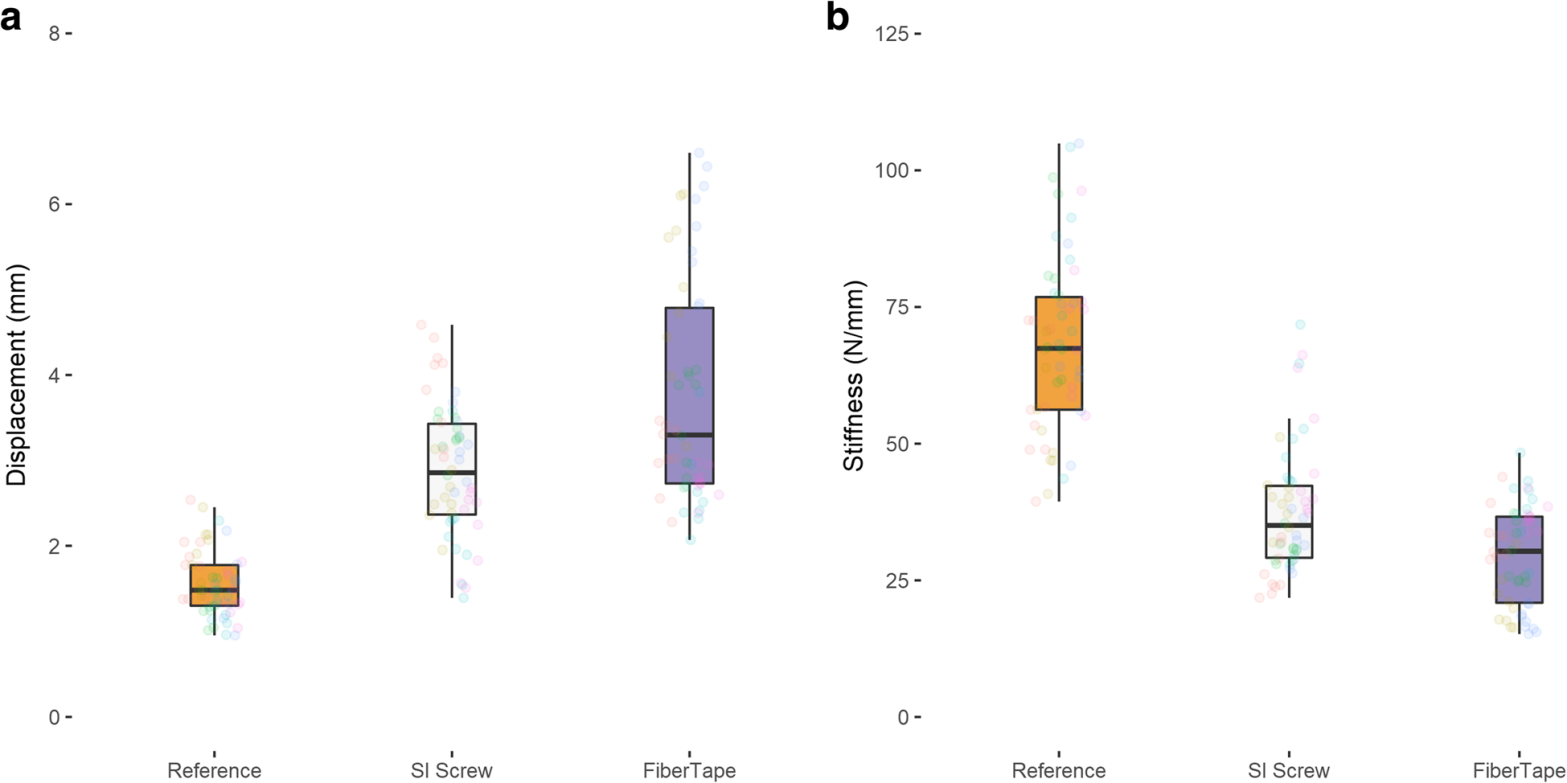
**a**, **b** Average displacement and stiffness measurements at the anterior fracture site

Considering the anterior fracture site, the FiberTape® again demonstrated similar displacement (3.8 ± 1.3 mm) and stiffness (29.5 ± 9.0 N/mm) compared to the sacroiliac screw (displacement 2.9 ± 0.8 mm, *P* = 0.2196; stiffness 37.5 ± 11.5 N/mm, *P* = 0.0711) (Table 3) (Fig. 3).

## Discussion

The treatment of incomplete posterior pelvic ring fractures (AO type B2.1) can be performed by various conservative and surgical procedures [20–23]. However, conservative therapy shows significantly longer immobility and an increased level of pain [24]. The most frequently surgical method used is percutaneous sacroiliac screw fixation [20]. The advantages are the percutaneous approach with a short operation time and minimal soft tissue injury [20, 25–27]. However, this type of osteosynthesis also has various risks. Due to the complex geometry of the pelvis, a high degree of expertise is required to ensure exact placement of the screw [28]. Osteosynthesis performed with conventional x-ray often show an insufficient representation of anatomical structures, especially in obese patients, in patients with intestinal gas overlay or when positioning a screw in the S2 segment. Thus, screw malposition can be seen in 2 to 68% of patients treated with S1/S2 screw fixation, whereas neurologic symptoms are seen in 0.5 to 7.9% [29, 30]. Methods such as plate osteosynthesis or internal fixation with pedicle screws have a significantly higher morbidity due to the open approach or often lead to irritation of the soft tissue, which can cause problems such as lesions of the skin or pain in the area of the iliac bone [23, 25].

Minimally invasive posterior tension banding with suture tape through the posterior superior iliac spines is a newly introduced method for stabilizing the posterior pelvic ring. This procedure is a minimally invasive fixation method with a sufficient posterior fracture stabilization without implanting any soft tissue disturbing components such as pedicle screw constructs. In addition, the extra-sacral position avoids an injury to the sacral nerve roots. Compared to SI screw fixation, tension banding does not compromise the intact SI joint. We assume that this semi-rigid fixation method also allows physiological mobility in the SI joint, so that removal of osteosynthesis implants is not necessary and there is no danger of stiffening of the joint. Positioning of the drill holes in the posterior superior iliac spine is usually easy to determine from the anatomical landmarks or to visualize in X-rays. This means that no special equipment such as 3D x-ray or navigation system is required to ensure correct positioning of the implants. We think that the radiation exposure and the surgical demands are also lower in this minimally invasive procedure compared to standard sacroiliac screw fixation.

Our biomechanical results showed no significant difference between screw fixation and minimally invasive posterior pelvic tension banding considering the dislocation of both the anterior and the posterior fracture gap under cyclic loading. Using the FiberTape®, the dislocation of the posterior pelvic ring was 0.2 mm higher (3.2 mm ± 0.9) than with the sacroiliac screw (3.0 mm ± 0.5), however not significantly. Considering the anterior pelvic fracture, the dislocation using the FiberTape® was 0.9 mm higher than the sacroiliac screw fixation, but again not significantly. With a resolution of the ultrasound measuring system of 0.1 mm, we showed that the stabilization of the posterior pelvic ring with a minimally invasive tension banding achieves a comparable stability as a sacroiliac screw. The effect of a higher dislocation of the anterior pelvic fracture as seen in the FiberTape® group could also be seen in conservatively treated pelvic fractures type B 2.1 without any problems in bone healing of the anterior pelvic ring in patients under 65 years [31]. We therefore assume that the slightly increased dislocation at the anterior pelvic ring using tape suture for posterior pelvic ring stabilization is not clinically relevant. The incomplete sacral fracture is good controlled with the extra-sacral implanted tape suture. But we think in higher unstable and dislocated fractures especially at the anterior pelvic ring and complete sacral fractures, the tape suture could not prevent opening of the anterior sacral fracture, as well as the anterior pelvic ring fracture. Thus we, think that our described method does work best in incomplete sacral fractures. Pain control in the sacral area is the most relevant clinical aim in incomplete posterior pelvic ring fracture or insufficiency fractures [32]. In our opinion, a tape suture on patients with incomplete posterior pelvic fracture could be a feasible minimally invasive treatment option for alleviating pain.

In the results presented here, we showed that the stabilization of the posterior pelvic ring was sufficiently achieved by minimally invasive dorsal tension using tape suture.

## Conclusion

The newly presented osteosynthesis of the posterior superior iliac spine using FiberTape® shows promising results for the stabilization of the posterior pelvic ring in AO Type B2.1 lateral compression fractures of the pelvis when compared to sacroiliac screw osteosynthesis. Further advantages of the technique presented here are the lack of the necessity of implant removal, lower surgical demands, and lower risk of injury to relevant neural or vascular structures of the patient.

## Data Availability

The authors confirm that the data supporting the findings of this study are available within the article and its supplementary materials.

## Abbreviations

3D: 3-dimensional
AO: Arbeitsgemeinschaft Osteosynthese, Working Group Osteosynthesis
Fig.: Figure
FöFoLe: Förderprogramm für Forschung und Lehre, support program for research and teaching
GmbH: Gesellschaft mit beschränkter Haftung, private corporation with limited liability
No.: Number
qCT: Quantitative computed tomography
SD: Standard deviation
SI: Sacroiliac
TIFI: Transiliac internal fixator

## References

[1] Krappinger Dietmar, Kaser Verena, Kammerlander Christian, Neuerburg Carl, Merkel Anke, Lindtner Richard A. (2019). Inter- and intraobserver reliability and critical analysis of the FFP classification of osteoporotic pelvic ring injuries. Injury.

[2] Li S, Meng X, Li W, Sun Z, Wang X, Qi H, Wei S, Zhou D (2018). Effects of minimally invasive plate-screw internal fixation in the treatment of posterior pelvic ring fracture. Exp Ther Med.

[3] Salari P, Cannada LK, Moed BR (2015). Do asymptomatic patients have normal function after percutaneous fixation of the posterior pelvic ring? A case-control pilot study. J Orthop Surg Res.

[4] Lee S-W, Kim W-Y, Koh S-J, Kim Y-Y (2017). Posterior locked lateral compression injury of the pelvis in geriatric patients: an infrequent and specific variant of the fragility fracture of pelvis. Arch Orthop Trauma Surg.

[5] Höch A, Özkurtul O, Pieroh P, Josten C, Böhme J (2017). Outcome and 2-year survival rate in elderly patients with lateral compression fractures of the pelvis. Geriatr Orthop Surg Rehabil.

[6] Beckmann JT, Presson AP, Curtis SH, Haller JM, Stuart AR, Higgins TF, Kubiak EN (2014). Operative agreement on lateral compression-1 pelvis fractures. A survey of 111 OTA members. J Orthop Trauma.

[7] Yinger K, Scalise J, Olson SA, Bay BK, Finkemeier CG (2003). Biomechanical comparison of posterior pelvic ring fixation. J Orthop Trauma.

[8] Giráldez-Sánchez MA, Lázaro-Gonzálvez Á, Martínez-Reina J, Serrano-Toledano D, Navarro-Robles A, Cano-Luis P, Fragkakis EM, Giannoudis PV (2015). Percutaneous iliosacral fixation in external rotational pelvic fractures. A biomechanical analysis. Injury.

[9] Iorio JA, Jakoi AM, Rehman S (2015). Percutaneous sacroiliac screw fixation of the posterior pelvic ring. Orthop Clin North Am.

[10] Pieske O, Landersdorfer C, Trumm C, Greiner A, Wallmichrath J, Gottschalk O, Rubenbauer B (2015). CT-guided sacroiliac percutaneous screw placement in unstable posterior pelvic ring injuries: accuracy of screw position, injury reduction and complications in 71 patients with 136 screws. Injury.

[11] van den Bosch EW, van Zwienen CMA, van Vugt AB (2002). Fluoroscopic positioning of sacroiliac screws in 88 patients. J Trauma.

[12] Kerschbaum M, Hausmann N, Worlicek M, Pfeifer C, Nerlich M, Schmitz P (2017). Patient-related outcome of unstable pelvic ring fractures stabilized with a minimal invasive screw-rod system. Health Qual Life Outcomes.

[13] Vigdorchik JM, Jin X, Sethi A, Herzog DT, Oliphant BW, Yang KH, Vaidya R (2015). A biomechanical study of standard posterior pelvic ring fixation versus a posterior pedicle screw construct. Injury.

[14] Regauer M, Mackay G, Lange M, Kammerlander C, Böcker W (2017). Syndesmotic Internal Brace TM for anatomic distal tibiofibular ligament augmentation. World J Orthop.

[15] Gilmer BB, Crall T, DeLong J, Kubo T, Mackay G, Jani SS (2016). Biomechanical analysis of internal bracing for treatment of medial knee injuries. Orthopedics.

[16] Ono Y, Joly DA, Thornton GM, Lo IKY (2018). Mechanical and imaging evaluation of the effect of sutures on tendons: tape sutures are protective to suture pulling through tendon. J Shoulder Elbow Surg.

[17] Nothofer W, Thonke N, Neugebauer R (2004). Die Therapie instabiler Sakrumfrakturen bei Beckenringbruechen mit dorsaler Sakrumdistanzosteosynthese. Der Unfallchirurg.

[18] Soin SP, Knight TA, Dinah AF, Mears SC, Swierstra BA, Belkoff SM (2009). Suture-button versus screw fixation in a syndesmosis rupture model: a biomechanical comparison. Foot Ankle Int.

[19] Mcdonald E., Theologis A. A., Horst P., Kandemir U., Pekmezci M. (2014). When do anterior external or internal fixators provide additional stability in an unstable (Tile C) pelvic fracture? A biomechanical study. European Journal of Trauma and Emergency Surgery.

[20] van den Bosch EW, van Zwienen CMA, Hoek van Dijke GA, Snijders CJ, van Vugt AB (2003). Sacroiliac screw fixation for tile B fractures. J Trauma.

[21] Krappinger D, Larndorfer R, Struve P, Rosenberger R, Arora R, Blauth M (2007). Minimally invasive transiliac plate osteosynthesis for type C injuries of the pelvic ring: a clinical and radiological follow-up. J Orthop Trauma.

[22] Kobbe P, Hockertz I, Sellei RM, Reilmann H, Hockertz T (2012). Minimally invasive stabilisation of posterior pelvic-ring instabilities with a transiliac locked compression plate. Int Orthop.

[23] Wang H, Fu Y-H, Ke C, Zhuang Y, Zhang K, Wei X, Li Z, Lei J-L, Zhang B-F, Liu P (2018). Minimally invasive stabilisation of posterior pelvic ring instabilities with pedicle screws connected to a transverse rod. Int Orthop.

[24] Quinn Robert H., Sanders James O., Brown Gregory Alexander, Murray Jayson, Pezold Ryan (2016). The American Academy of Orthopaedic Surgeons Appropriate Use Criteria on the Management of Anterior Cruciate Ligament Injuries. The Journal of Bone and Joint Surgery.

[25] Routt ML, Simonian PT, Mills WJ (1997). Iliosacral screw fixation: early complications of the percutaneous technique. J Orthop Trauma.

[26] Osterhoff G, Ossendorf C, Wanner GA, Simmen H-P, Werner CML (2011). Posterior screw fixation in rotationally unstable pelvic ring injuries. Injury.

[27] Chalidis B, Stengel D, Giannoudis PV (2007). Early excision and late excision of heterotopic ossification after traumatic brain injury are equivalent: a systematic review of the literature. J Neurotrauma.

[28] Templeman D, Schmidt A, Freese J, Weisman I (1996). Proximity of iliosacral screws to neurovascular structures after internal fixation. Clin Orthop Relat Res.

[29] Behrendt D, Mütze M, Steinke H, Koestler M, Josten C, Böhme J (2012). Evaluation of 2D and 3D navigation for iliosacral screw fixation. Int J Comput Assist Radiol Surg.

[30] Arand Markus, Kinzl Lothar, Gebhard Florian (2004). Computer-Guidance in Percutaneous Screw Stabilization of the Iliosacral Joint. Clinical Orthopaedics and Related Research.

[31] Höch A, Schneider I, Todd J, Josten C, Böhme J (2018). Lateral compression type B 2-1 pelvic ring fractures in young patients do not require surgery. Eur J Trauma Emerg Surg.

[32] Sanders D, Fox J, Starr A, Sathy A, Chao J (2016). Transsacral-transiliac screw stabilization: effective for recalcitrant pain due to sacral insufficiency fracture. J Orthop Trauma.

